# Quantifying the link between retinal performance and the optomotor response

**DOI:** 10.1016/j.cub.2025.07.007

**Published:** 2025-07-25

**Authors:** Patrício Simões, José Moya-Díaz, Leon Lagnado

**Affiliations:** 1 Sussex Neuroscience, School of Life Sciences, University of Sussex, Sussex, Brighton BN19QG, UK

## Abstract

Visually driven behaviors continuously adjust as the animal experiences different external environments or internal states. To understand the neural basis of this plasticity, we must quantify the relation between changes in the operation of circuits in the visuo-motor pathway and the behavioral output. Here, we use larval zebrafish to measure how modulation of the retina impacts the optomotor response (OMR). We find that diurnal changes in the contrast dependence of the OMR are linearly dependent on changes in the rate at which information about contrast is transmitted across the population of bipolar cells: when synaptic information rates increased 4-fold, the contrast gain of the behavior increased 2.4-fold. The energetic cost of the improvement in retinal performance was offset by the circuit switching to a state in which each vesicle transmitted 2–4 times as much information at any given release rate. These results demonstrate that retinal noise limits a behavior driven by cone photoreceptors, not just at threshold but also across a range of contrasts found in natural stimuli. Adjustment of the retinal circuit between operating regimes of higher and lower information efficiency also demonstrates a previously unrecognized aspect of modulation by internal state that we propose reflects a trade-off between the energetic cost of retinal computation and the ecological benefits of visually driven behaviors at different stages of the solar cycle. This study supports the idea that information measures will be useful in investigating links between the operation of neural circuits and behavior.

## Graphical abstract

**Graphical abstract:**
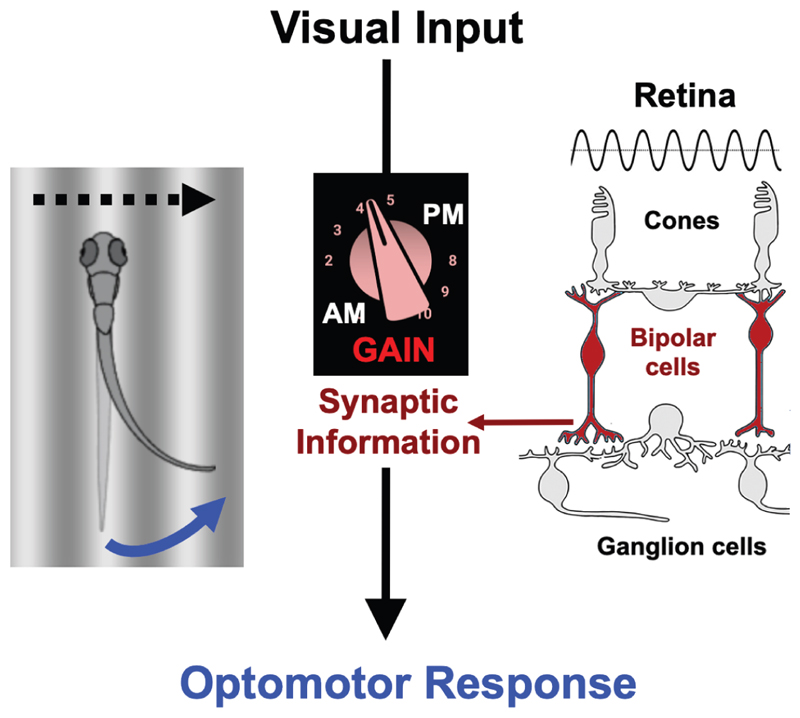

## Introduction

Visually driven behaviors are not static but continuously adjust as the animal experiences different external environments, such as daily fluctuations in light levels, or internal states, such as attention, arousal, or circadian rhythms.^1–3^ This plasticity is thought to involve modulation at multiple stages of the pathways linking the retina to motor circuits, but we do not understand the relation between neural activity at any one of these stages and the final behavioral output. Here, we use zebrafish to quantify how modulation of a fundamental retinal processing task—detection of contrast—impacts the optomotor response (OMR). The OMR is an innate behavior where the animal moves in the direction of whole-field visual motion to stabilize its position relative to the visual world. The OMR operates in animals ranging from flies^4^ to mammals,^5^ and in diurnal species it is modulated both by an intrinsic circadian clock and the solar cycle.^6–8^

A classical approach for investigating how signals in the retina determine perceptual responses has been to observe both at the thresholds of detection. When a toad is hunting worms under dim starlight, individual rod photoreceptors reliably signal each photon, but random thermo-isomerization of rhodopsin generates background noise that determines the strength of the dimmest stimulus that can be reliably detected.^9–12^ Thresholds for cone vision are also limited by noise, but the dominant sources are thought to be in synaptic compartments of cones and bipolar cells.^13–15^ These analyses of detection thresholds provide a useful but limited picture of the link between retinal function and behavior. In daylight, natural scenes contain contrasts of 30% or more—far above the ~ 1–2% threshold for photopic vision in humans or mice.^16,17^ We now need to understand how the performance of the retina limits behavioral responses to the range of contrasts normally experienced during daylight vision.

Neural computations require energy, and this places fundamental constraints on the design and operation of sensory circuits.^18–20^ One approach to assessing the information efficiency of neurons has been to calculate the number of bits of information carried by spikes,^21,22^ but, in actuality, excitatory synaptic transmission has a greater cost. In cortical neurons, for instance, spikes account for ~22% of ATP consumption, whereas maintaining glutamatergic transmission accounts for ~58%.^18^ Quantifying bits per glutamatergic vesicle therefore provides more direct insights into the efficiency of a neural circuit as a computing device.^19,23^ We can now make such measurements in the retina of zebrafish by using the fluorescent reporter iGluSnFR to image the release of individual glutamatergic vesicles as they transmit the visual signal from bipolar cells to neurons of the inner retina.^24,25^ About half the neurons in the brain of larval zebrafish reside within the retina,^26^ providing a powerful model for investigating the links between modulation of circuit function, the costs of information processing, and behavior.

We focus on bipolar cells because they form a bottleneck in the flow of visual information, being the only connection between photoreceptors and the rest of the retinal circuit. Many of the most fundamental retinal computations are already evident in the output of bipolar cells, including contrast adaptation, motion detection, and orientation selectivity. Further, the output of all bipolar cells can be imaged in one location, the inner plexiform layer, whereas ganglion cells deliver the results of retinal computations to about 10 different retino-recipient areas, only a minority of which play a role in the OMR. The visual signals that bipolar cells transmit to the inner retina reflect neuromodulatory changes in the operation of the circuit, allowing us to ask how these impact the OMR. To achieve this, we manipulated two neuromodulatory systems, dopamine and substance P, which have opposing actions on contrast gain during the diurnal cycle^27^: dopamine acts through D1 receptors on bipolar cell terminals to increase gain whereas substance P acts through the neurokinin 1 (NK1) to reduce it. In the morning, substance P is much more active than dopamine, and this reverses in the afternoon through a “push-pull” interaction of the two systems, likely through amacrine cells releasing substance P at specific connections with dopaminergic amacrine cells.

We find that between the morning and afternoon phases of the diurnal cycle, changes in the contrast dependence of the OMR are linearly dependent on changes in the rate at which information about contrast is transmitted across the population of bipolar cells. This modulation is strong—when synaptic information rates increased 4-fold, the contrast gain of the OMR increased 2.4-fold. The energetic cost of the improvement in retinal performance was offset by the circuit switching to a regime in which each vesicle transmitted 2–4 times as much information at any given release rate. This study reveals how changes in retinal signals and noise limit performance of a behavior driven by cone photoreceptors, not just at threshold but also across a range of contrasts found in natural stimuli. Plasticity of the OMR is accompanied by the retinal circuit adjusting between operating regimes of higher and lower information efficiency.

## Results

A fundamental property of a visual stimulus is its strength, quantified as contrast. Here, we compare the contrast dependence of the OMR with the information about this same stimulus parameter transmitted by retinal bipolar cells.

### Diurnal modulation of the OMR and retinal function

**Figure 1:**
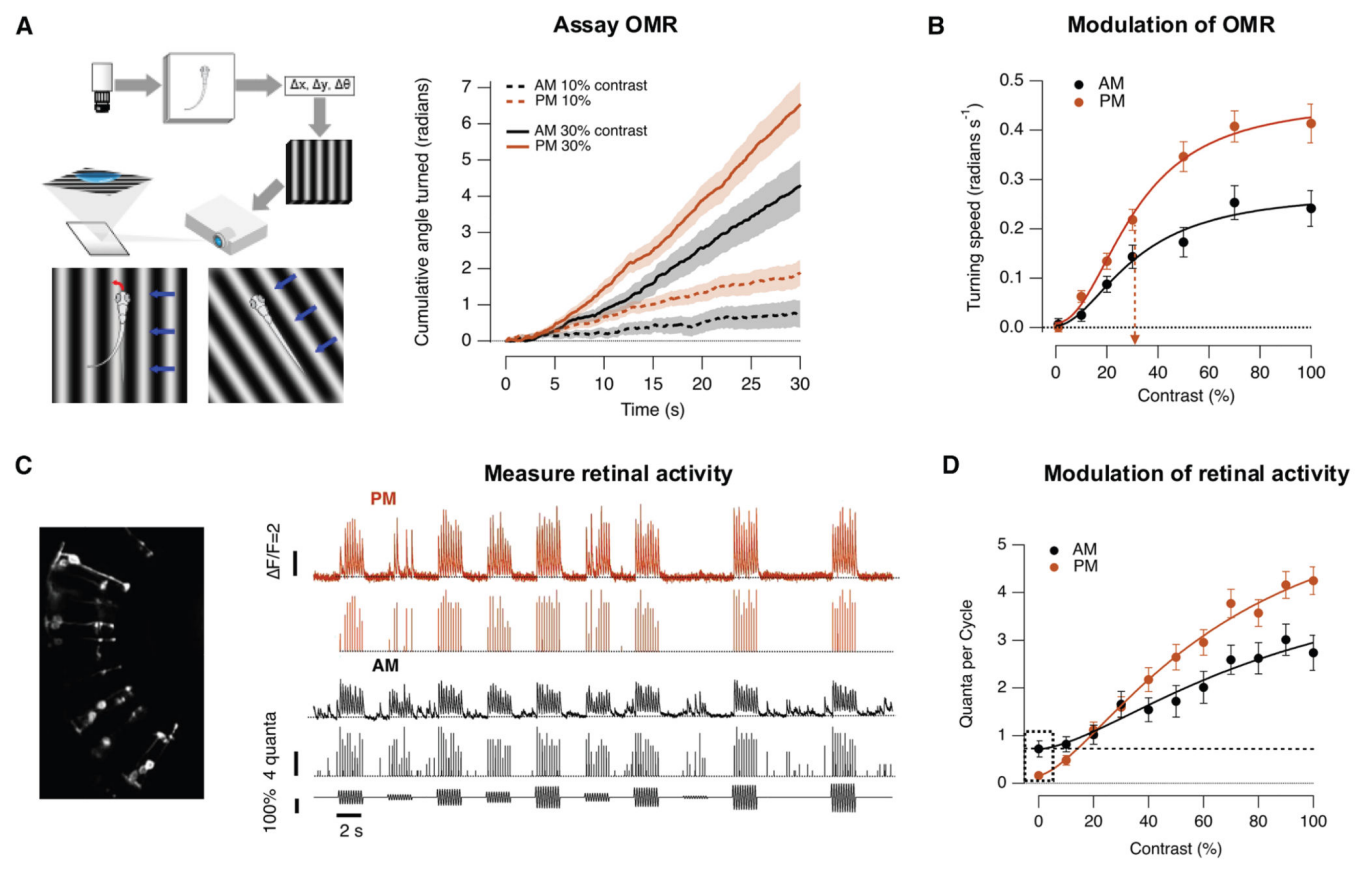
Comparing the OMR and retinal function (A) Left: behavioral setup. Free-swimming fish are monitored with a camera while moving gratings are presented. The larvae position (Δx, Δy) and heading (Δθ) are extracted to update the visual motion direction of the gratings 90^◦^ relative to the body axis (larva image courtesy of Gil Costa in SciDraw). Right: the cumulative turning angle in response to contrasts of 10% and 30% during the morning (a.m., zeitgeber time [ZT]: 1 h, black) and afternoon (p.m., ZT: 7 h, brown). (B) Behavioral CRF in the a.m. (*n* = 67 fish) and p.m. (*n* = 93 fish). The strength of the behavior is quantified as the average turning speed (the quotient between the final cumulative angle and trial duration). Curves are Hill plots, both with half-maximal response at C_1/2_ = 31%. The curve at p.m. is the curve at a.m. multiplied by 1.7. a.m.: h = 1.9 ± 0.8, C_1/2 =_ 31 ± 8%; p.m.: h = 2.1 ± 0.4, C_1/2 =_ 31 ± 3%. (C) Left: *in vivo* imaging of iGuSnFR in bipolar cells. Right: examples of iGluSnFR signals from individual synapses in the morning (black) and afternoon (brown). The top trace shows the iGluSnFR signal and the lower trace the estimated number of vesicles released within an exocytic event. Modulation of light intensity at bottom. Note that spontaneous events occur in the absence of stimulation, most obviously in the morning. (D) Retinal CRF in the morning and afternoon. The strength of the synaptic response is quantified as the number of vesicles released per cycle of the stimulus. a.m.: h = 1.6 ± 0.9, C_1/2_ = 45 ± 14% (*n* = 31 synapses; measured between baseline and maximum response). p.m.: h = 1.5 ± 0.3, C_1/2_ = 35 ± 5% (*n* = 90 synapses). Note that spontaneous release in the absence of a stimulus (dashed box) was higher in the morning (3.6 ± 0.4 vesicles s^−1^) than the afternoon (0.8 ± 0.3 vesicles s^−1^). See also Figure S1.

The OMR was assayed in free-swimming zebrafish larvae where vision is driven exclusively by cones.^28,29^ A closed-loop paradigm was used where a sinusoidal grating drifted orthogonally to the longitudinal axis of the fish^30^ (Figures 1A and S1). The fluctuations in intensity experienced by any individual bipolar cell in the retina were close to spatially uniform (STAR Methods), but population processing across both eyes biased the fish’s turning decisions such that more turns were made in the direction of whole-field motion. Turning bias was measured by continuously tracking head direction for 30–60 s and calculating the average angular velocity (Figure 1B), from which a behavioral contrast-response function (CRF) was constructed for 2-h time windows in the morning (a.m., centered on zeitgeber time 1 h) and afternoon (p.m., zeitgeber time 7 h; Figure 1A). These periods were chosen because the contrast sensitivity of the retina shows a distinct peak in the afternoon phase of the diurnal cycle.^6,25^ The OMR was strongly potentiated in the afternoon, increasing by a factor of 1.7 compared with the morning, without any significant change in contrast sensitivity (C_1/2_ = 31%; Figure 1B).

In a parallel set of experiments, the synaptic output from bipolar cells was imaged using iGluSnFR, and this was also amplified in the afternoon.^25,27^ These neural CRFs were measured using a full-field stimulus at the same frequency and mean luminance used to investigate the behavior. An example of iGluSnFR signals from an individual OFF synapse is shown in Figure 1C, together with the estimated number of vesicles associated with each release event (STAR Methods). When averaged across all types of responsive bipolar cells (*n* = 151), a stimulus of 100% contrast released an average of 4.3 ± 0.3 vesicles per stimulus cycle in the afternoon compared with 2.7 ± 0.3 vesicles per cycle in the morning. A second notable change was a decrease in spontaneous synaptic noise from 3.6 ± 0.4 vesicles s^−1^ in the morning compared with 0.8 ± 0.3 vesicles s^−1^ in the afternoon (Figure 1D). This modulation is stronger in OFF synapses compared with ON.^25^ Potentiation of the OMR was therefore correlated with increased amplitude and reduced variability of signals transmitted through the population of bipolar cells, at least to a first approximation.

### The OMR is adjusted by retinal neuromodulators

Sensorimotor behaviors are shaped by multiple stages of sensory processing followed by motor execution, and any of these stages can exhibit plasticity. Different brain regions can generate their own intrinsic rhythms^31^ and we cannot, therefore, assume that diurnal changes in retinal function are the primary driver of changes in the OMR. The neuromodulator dopamine can increase the number of motor neurons recruited during an OMR swim bout, causing a stronger turn through a larger angle,^32^ so a second possibility is that circadian control is exerted at the motor stage.

**Figure 2:**
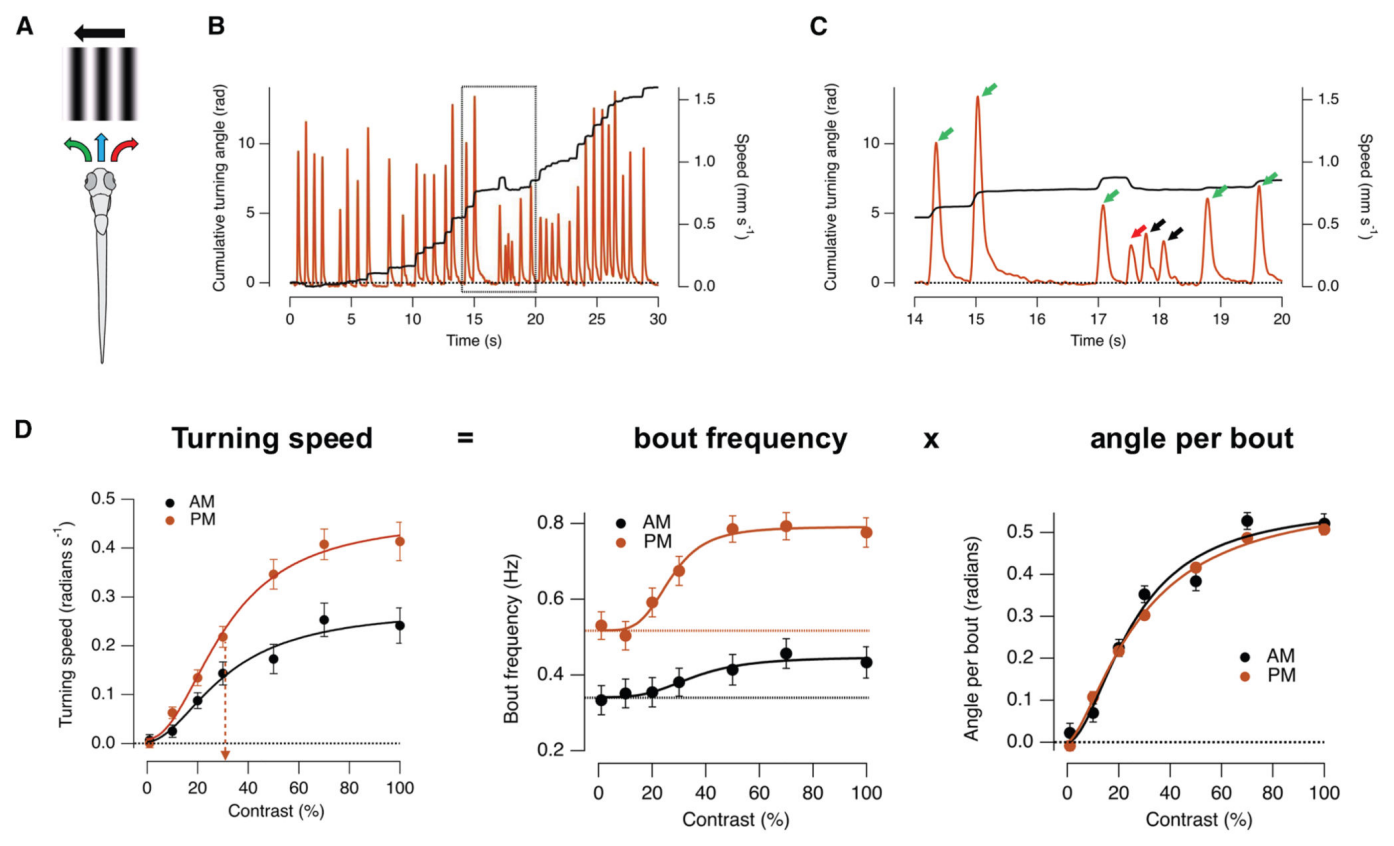
Diurnal modulation of the OMR did not involve computations that map stimulus contrast to motor commands (A) “Correct” swimming bouts are defined as responses in which the fish turns in the direction of the moving grating so to stabilize its position relative to global motion (green arrow). “Errors” are bouts in which fish turns in a direction opposed to the grating (red). Other bouts (termed scoots) are forward and do not involve a significant turn (blue). (B) Example of kinematic analysis in response to 100% contrast. Brown: instantaneous speed of motion. Black: cumulative angle turned. (C) Sample from B on expanded timescale. Scoots (black), “correct” turns (green), and “errors” (red) are arrowed. (D) Turning speed (left, from Figure 1B) factorized into the product of the frequency of swimming bouts (center) and the average angle turned per bout (right). In the absence of a stimulus, fish were twice as likely to make swims in the afternoon compared with the morning, but these were mainly forward motions (scoots), and turns left or right were equally probable, reflecting a generally more active state. Potentiation of the OMR in the afternoon was caused by an increase in the frequency of swim bouts in response to a stimulus but *without* any significant change in the angle moved at any given contrast.

To test whether diurnal modulation of the OMR reflected execution of the swim command, we measured the amplitude of individual swim bouts (Figures 2A–2C) and factorized turning speed into the product of the frequency of swimming bouts and the average angle turned per bout (Figure 2D). In the absence of a stimulus, fish were twice as likely to make swims in the afternoon compared with the morning, but these were mainly forward motions (“scoots”), and turns left or right were equally probable, reflecting a generally more active state. Potentiation of the OMR in the afternoon was caused by an increase in the frequency of swim bouts in response to a stimulus (Figure 2D, center) but *without* any significant change in the angle moved at any given contrast (Figure 2D, right). In other words, once stimulus contrast was computed, circuits that map the result to motor commands operated in the same way in the morning and afternoon.

**Figure 3:**
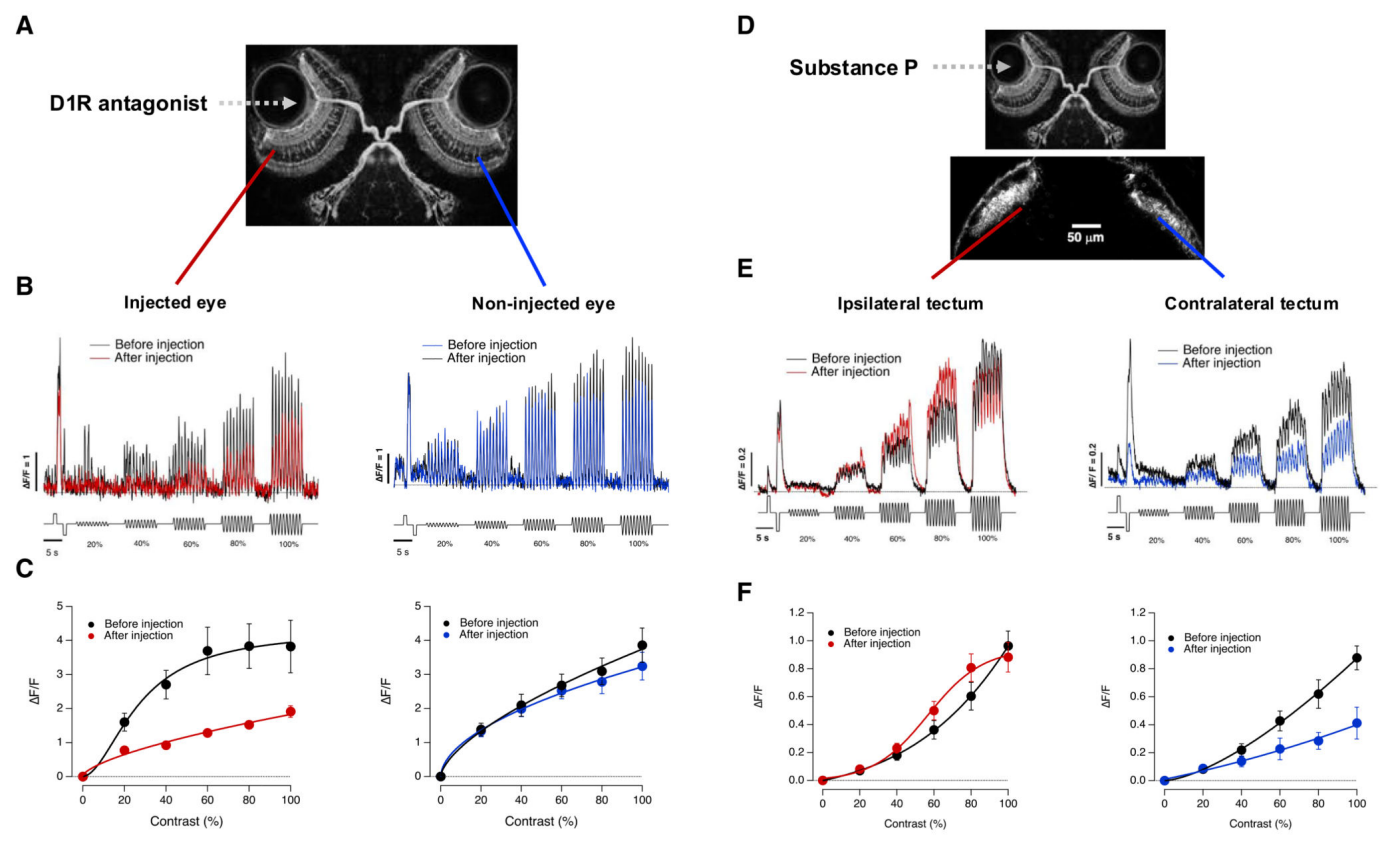
Manipulation of retinal function (A) The D1R antagonist SCH23390 (20 nM) was injected into one eye of a fish in the afternoon (image courtesy of Steve Wilson and the Wellcome Image Library). (B) SyGCaMP6f signals generated by stimuli of varying contrasts measured from bipolar cells before and after injection (left) compared with the non-injected eye (right). Example traces from one fish. (C) Averaged CRFs from experiments in B. Activity in the injected eye was significantly suppressed (ANOVA, F = 102.8, *p* < 0.001, *n* = 7 synapses) but the non-injected eye was unaffected (ANOVA, F = 1.4, *p* = 0.24, *n* = 7 synapses). (D) Substance P (20 nM) was injected into one eye in the afternoon. The superficial layers of each optic tectum were imaged in fish expressing the synaptic calcium reporter mGCaMP6f in RGCs under control of the *isl2b* promoter. (E) The average mGCaMP6f signal in each optic tectum before (black) and within 20 mins (red or blue) of the injection. Example traces from one fish. (F) Averaged CRFs from experiments in (E). Output of the injected eye to the contralateral tectum was suppressed (right; ANOVA, F = 20.1, *p* < 0.001, *n* = 11 synapses) but signals in ipsilateral tectum were unaffected (ANOVA, F = 1.16, *p* = 0.16, *n* = 13 synapses). See also Figure S2.

To test whether the retina was the primary determinant of diurnal changes in the OMR, we manipulated it by injecting agonists and antagonists of receptors for dopamine (D1R) and substance P (NK1R) into the eye. Although dopamine “pushes” the retina to transmit information at higher rates in the afternoon, substance P acts in antiphase to suppress dopamine signaling and “pull-down” information transmission in the morning.^27^ A potential problem, however, is that these receptors are also present in other parts of the brain. We therefore tested whether drugs exerted effects beyond the injected eye by measuring activity in two other parts of the brain: bipolar cells in the non-injected eye, using SyGCaMP6f (Figures 3A–3C), and RGC axon terminals in the ipsilateral tectum, using mGCaMP6f (Figures 3D–3F). Bipolar cell activity in the eye injected with the D1R antagonist SCH233090 (estimated final concentration of 20 nM) in the afternoon was significantly suppressed within the first 20–30 mins after injection, but the non-injected eye was unaffected (Figures 3A–3C). The RGC output in the contralateral tectum from the eye injected with substance P (20 nM) in the afternoon was suppressed, but the RGC output in the ipsilateral tectum (from the non-injected eye) was unaffected (Figures 3D–3F). Similar results were obtained in all eleven animals tested in this way, and injection of the carrier alone had no significant effect on the OMR (Figure S2). These manipulations of neuromodulator receptors therefore caused an acute and local change in contrast processing in the retina.

**Figure 4:**
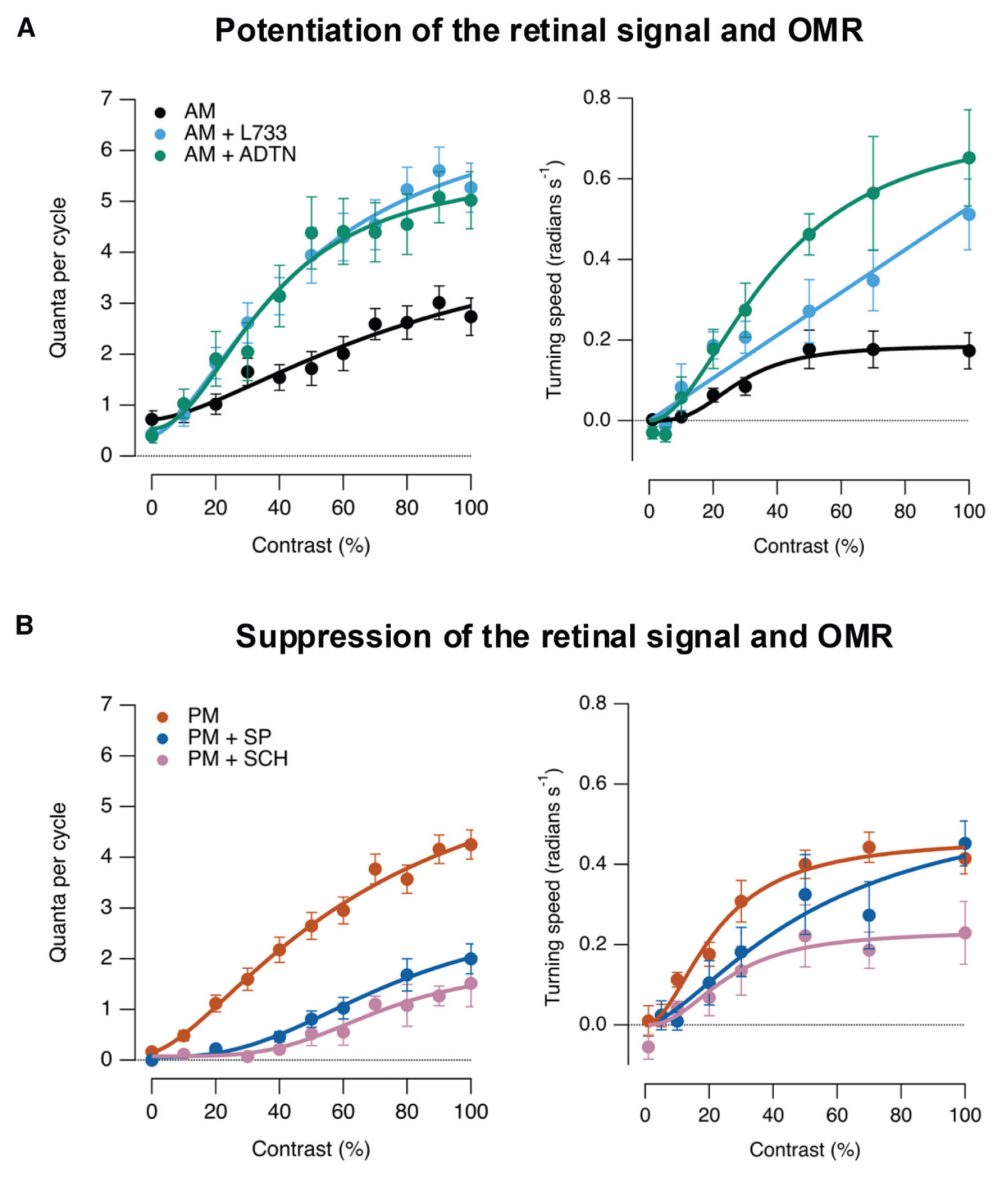
The OMR is regulated by the retinal neuromodulators substance P and dopamine (A) Potentiation of the retinal signal and OMR. Neural (left) and behavioral (right) CRFs in the morning, measured as in Figure 1. The effects of pharmacology on the quantal release of bipolar cell synapses were compared between controls (black) and L733,060 (cyan; ANOVA: F = 121.17, *p* < 0.001; *n* = 23) or ADTN (green; ANOVA: F = 73.27; *p* < 0.001, *n* = 17). The effects of pharmacology on the behavioral performance were compared between controls (black) and L733,060 (cyan; ANOVA: F = 28.43, *p* < 0.001; *n* = 25) or ADTN (green; ANOVA: F = 47.03, *p* < 0.001; *n* = 21). (B) Suppression of the retinal signal and OMR. Neural (left) and behavioral (right) CRFs in the afternoon. The effects of pharmacology on the quantal release of bipolar cells synapses were compared between controls (orange) and substance P (blue; ANOVA: F = 77.9, *p* < 0.001; *n* = 25) or SCH23390 (purple; ANOVA: F = 67.65, *p* < 0.001; *n* = 20). The effects of pharmacology on the behavioral performance were compared between controls (orange) and substance P (blue; ANOVA: F = 6.37, *p* = 0.013; *n* = 24) or SCH23390 (purple; ANOVA: F = 28.05, *p* < 0.001; *n* = 24). Curves fitted to points are Hill functions with the purpose of highlighting that changes in CRFs were not simply multiplicative. For OMR measurements, injections were made in both eyes.

To test how changes in retinal function affected the OMR, we manipulated dopamine or substance P signaling in both eyes. First, we imposed *increases* in retinal gain in the morning, either by activating D1 receptors using ADTN or by antagonizing NK1 receptors using L733060 (Figure 4A, left). In both cases, the OMR was potentiated, although the magnitude of the change depended on the contrast at which it was measured (Figure 4A, right). Conversely, two manipulations that *reduced* retinal gain in the afternoon (injection of substance P or antagonizing D1 receptors) also suppressed the OMR (Figure 4B). These results demonstrate that retinal processing limits the OMR across the range of contrasts found in natural stimuli, which, in turn, allows this behavior to be adjusted by modulators of retinal processing dopamine and substance P.^27^

### Synaptic information rates as a predictor of behavioral gain

**Figure 5:**
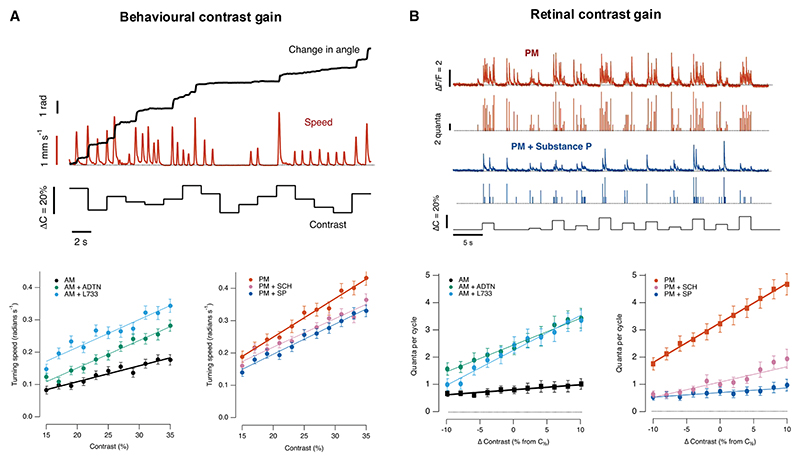
MCG as a metric to compare neural and behavioral performance (A) The OMR as a function of contrast. Top: example of a partial behavioral record. The grating (5 Hz) was presented at eleven different contrasts between 15% and 35% in a pseudo-random order and the speed of swim (brown) and cumulative change in angle (black) measured. Each contrast was presented 100 times. Bottom: average turning speed as a function of contrast in the three conditions in the morning (left) and afternoon (right). The slope of the lines fitted to the points is the MCG. a.m.: *n* = 14, a.m.+L733,060: *n* = 12, a.m. + ADTN: *n* = 15, p.m.: *n* = 14, p.m. + SP: *n* = 17; p.m. + SCH23390: *n* = 14 fish. (B) Synaptic transfer of the visual signal as a function of contrast. Top: example of iGluSnFR records and conversion to estimated number of quanta in the afternoon before (orange) and after (blue) injection of substance P. Again, 11 different contrasts (5 Hz) were presented in a pseudo-random order over the range ±10% around the synaptic C_1/2_. Bottom: the average number of quanta released per cycle of the stimulus in the three conditions in the morning (left) and afternoon (right). The slope of the lines fitted to the points is the MCG. a.m.: *n* = 25, a.m. + L733,060: *n* = 11, a.m. + ADTN: *n* = 21, p.m.: *n* = 65, p.m. + SP: *n* = 11; p.m. + SCH23390: *n* = 18 synapses.

How can we make a quantitative comparison between the changes in retinal function imposed experimentally and their consequences on the OMR? A classical approach for comparing neural and behavioral performance is to measure detection thresholds for each^11,13,33^ but here our aim was to make a comparison across a *range* of physiologically relevant stimulus strengths. To compare neural and behavioral performance with the same metric, we used the maximum contrast gain (MCG). Contrast gain is simply the slope of the CRF (Δresponse/Δcontrast), and its maximum is usually around the contrast generating the half-maximal response (C_1/2_). The MCG of the OMR was calculated using 11 contrasts over the range 15%–35% (Figure 5A). The contrast sensitivity of bipolar cell synapses varied more widely than the behavior, so, for each synapse, we first estimated C_1/2_ coarsely (0%–100% contrast in 11 steps) and then measured MCG more finely, again at ± 10% around C_1/2_ (Figure 5B). In each case, a linear fit to the CRF over a 20% range allowed relatively small changes in MCG to be quantified at the synapses of bipolar cells or the OMR.

**Figure 6:**
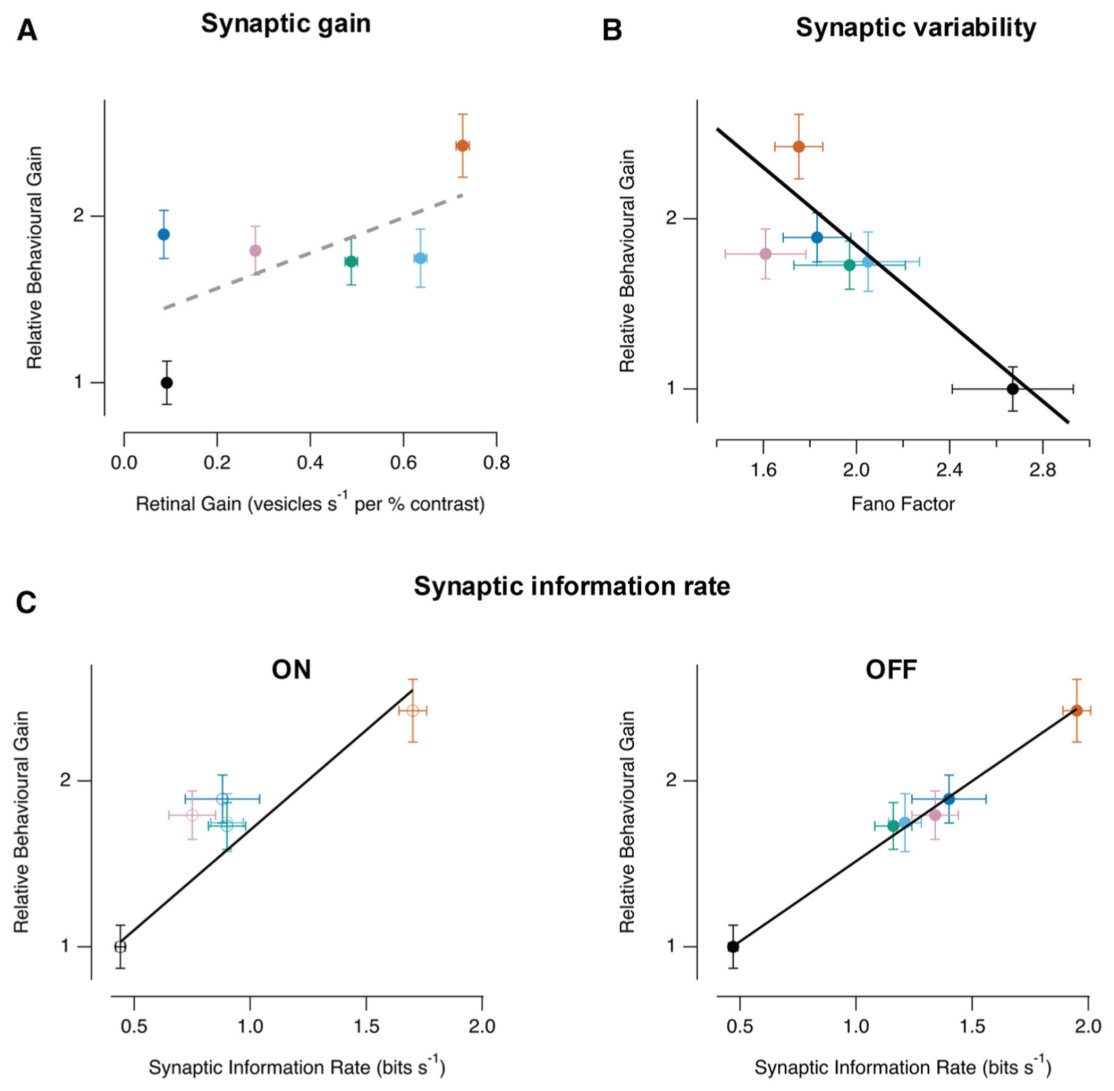
Metrics of retinal function as predictors of behavioral performance (A) Maximum contrast gain (MCG) of the OMR related to the MCG of the synaptic output from bipolar cells. Behavioral measurements have been normalized to the morning condition. Colors correspond to the six experimental conditions shown in Figures 4 and 5. There was no significant correlation between behavioral performance and retinal gain (Spearman rank correlation coefficient, Sr = 0.257143; critical value = 0.8 at p = 5%). (B) Relative MCG of the OMR as a function of the synaptic variability at contrasts ± 10% around C_1/2_. Linear correlation coefficient Pr = −0.84 (p = 0.04). (C) Relative MCG of the OMR as a function of the average synaptic information rate. ON synapses (n = 49): Pr = 0.91; p = 0.05. OFF synapses (n = 102), Pr = 0.97; p < 10^−7^. In the OFF channel, a 4-fold increase in synaptic information rates over the tested contrast range increased behavioral gain by a factor of 2.4.

Over the six different states of the retina shown in Figure 5A, the MCG of the OMR varied over a 2.4-fold range. How does this relate to the visual signal as it is transmitted from the population of bipolar cells? Surprisingly, there was no significant dependence on the average MCG of the population of bipolar cell synapses (Figure 6A; Spearman rank correlation coefficient, ρ = 0.257143; critical value = 0.8 at *p* = 5%). In other words, the amplitude of the visual signal as it is transmitted to the inner retina could not be used as a general predictor of the strength of the OMR.

Detection thresholds are dependent on noise in the retinal network.^11–15,34^ To test whether retinal noise limited the OMR over a range of behaviorally relevant contrasts, we quantified the variability of synaptic responses as the Fano factor, defined as the ratio of the variance of number of vesicles released per stimulus cycle divided by the mean.^25^ There was a significant negative correlation between the MCG of the OMR and the Fano factor averaged across the 20% range of contrasts, demonstrating that retinal noise also limited the behavior during daylight vision driven by cones (Figure 6B; linear correlation co-efficient Pr = 0.84; *p* < 0.04).

The combined effects of changes in gain and noise can be quantified using Information Theory, which in some sense measures the most fundamental function of a sensory system—the transfer of information about the environment. The third metric we used to relate changes in retinal function with the OMR was, therefore, the mutual information between the synaptic output of bipolar cells and the set of stimuli of 11 contrasts used to measure the MCG^25^ (see STAR Methods). Averaging measurements over a sample of 11–65 synapses in each condition, we found that the average rate at which information about stimulus contrast was transmitted was very strongly predictive of the MCG of the OMR, with almost perfect linear correlation through the OFF population of bipolar cells (Figure 6C: Pr = 0.97; *p* < 10^−7^). Performance of the OMR was more weakly correlated with information carried through the ON channel (Pr = 0.91; *p* < 0.05), consistent with evidence that this behavior is more sensitive to light-dark (OFF) transitions.^35^ The slope of the line through the data points in Figure 6C is of note: changing synaptic information rates by a factor of 4 altered the gain of the OMR by a factor of 2.4. These results demonstrate a strong and direct relation between the performance of the retinal circuit as an information-transmitting device and a behavior using that information.

### Diurnal changes in the efficiency regime of the retina

**Figure 7:**
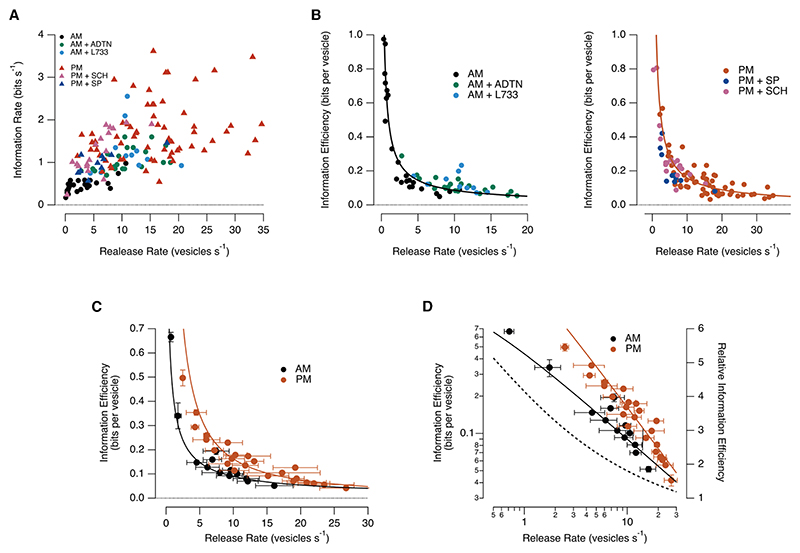
Diurnal changes in the efficiency regime of the retina (A) Rate of information transmission around C_1/2_. Scatterplot of information rate versus release rate for the six different conditions shown in Figure 5. (B) Information efficiency (E) falls as release rate (R) increases. Left: E as a function of R for each of the three conditions in the morning. Each point a single synapse. The power function fitted to the morning control is E = 0.49*R^−0.74^, and this also provided a good description of E when R was altered by the pharmacological manipulations. Right: as left but for the three conditions in the afternoon. The power function fitted to the morning control is E = 2.18*R^−1.12^. Again, efficiency at a given release rate was similar for the different conditions. (C) Diurnal change in the operating regime of the retina. Results from B after grouping all synapses in the morning and afternoon (each point 4–8 synapses). (D) Log-log plot of Figure 7C showing that the information efficiency in the afternoon was about twice that in the morning across a wide range of synaptic release rates. Dashed line corresponds to the ratio between E fit in the afternoon and the E fit in the morning.

The rate at which retinal ganglion cells transmit visual information as spikes increases with firing frequency, but the relation is sublinear so that efficiency, quantified as bits per spike, *falls* as neurons become more active.^22^ Similarly, the information efficiency of bipolar cell synapses, quantified as bits per vesicle, also fell as the information rate increased (Figures 7A and 7B). The relation between efficiency (E) and release rate (R) is plotted in Figure 7B for the three conditions in the morning (left) and afternoon (right), where each point represents a single synapse. In the morning, blocking NK1Rs or activating D1Rs shifted the population of bipolar cell synapses to higher release rates but with E falling as R^−0.75^ (Figure 7B, left). Conversely, blocking D1Rs or activating NK1Rs in the afternoon shifted synapses to lower release rates but with improved efficiency: in this case, E varied as R^−1.12^ (Figure 7B, right).

Closer inspection of Figure 7B suggests that, at a given release rate, the efficiency of transmission was not significantly affected by manipulations of D1Rs and NK1Rs (note the overlap of different colored points around the same release rate). To investigate this idea systematically, we pooled measurements in the morning and afternoon across conditions, as shown in Figure 7C. The resulting plot of information efficiency as a function of release rate shows that it was elevated in the afternoon compared with the morning, over and above the effects of changes in release rate. Efficiency increased 2-to 4-fold over a 10-fold range of release rates, and this diurnal difference only disappeared when more than 20 vesicles s^−1^ were released (dashed line in Figure 7D).

Diurnal modulation of the retina therefore impacted information transmission in two distinct ways. First, dopamine and substance P adjust the rate at which bipolar cells transmit information about contrast and, therefore, the efficiency measured as bits per vesicle (Figure 7B). Second, and over and above the actions of these neuromodulators, the circuit switches from a regime of low information efficiency in the morning to one of higher efficiency in the afternoon (Figures 7C and 7D). The energetic cost of improving the contrast gain of the OMR (Figure 6C) was therefore offset by increasing the efficiency with which the retina transmitted information about this property of the visual stimulus.

## Discussion

A general aim of contemporary neuroscience is to understand how computations in sensory circuits determine adaptive behaviors. Here, we have quantified the relationship between a fundamental visual processing task, detection of temporal contrast, and the OMR, using a range of conditions that alter both the function of the retina and the behavior (Figure 1). We find that diurnal modulation of the OMR primarily reflects adjustments on the sensory side of the pathway (Figure 2), dependent on the actions of two key neuromodulators—dopamine and substance P (Figures 3, 4, and 5). The information that bipolar cells transmit to the inner retina about stimulus contrast powerfully determines the strength of the behavior: a 4-fold increase in the rate of information transmission increased the contrast gain of the behavior by a factor 2.4 (Figure 6). Diurnal changes in the state of the retinal circuit also adjusted the cost of transmitting the results of this computation across synapses (Figure 7).

### Retinal information and noise

Identifying brain regions and cell types involved in a behavior is often done by ablation or optogenetics.^36,37^ In zebrafish, for instance, laser ablation of the retino-recipient area AF7 impairs prey detection,^38^ whereas optogenetic activation of neurons in the pretectum can initiate hunting.^39^ Our aim here has been to go beyond categorical findings, such as “this RGC subtype is involved in this response,” to understand the *quantitative* relationship between circuit performance and motor output. This task requires a well-defined computation and a metric of how well it is carried out. The results demonstrate that Shannon information can be a particularly useful metric in relating changes in the performance of a neural circuit to the plasticity of a behavior in which it is involved.^40–42^

A key advantage of information measures is that they take into account all the statistical properties of the system, including the amplitude and variability of signals, to provide an absolute measure of performance without reference to any underlying model of the way the system operates—or even the nature of the signal.^41,43^ In the retina, as in any neural circuit, information is continuously transformed between electrical and chemical forms. Electrical signals (graded or spikes) do not cross the synaptic junctions between neurons; that requires the electrical signal to activate (or, in some cases, suppress) the release of neurotransmitter. The electrical->chemical transformation of information at the synapse will necessarily involve a loss of information because of noise introduced by the stochastic nature of vesicle release.^44^ The next transformation, chemical->electrical, will lose information because of noise introduced by the chattering of ion channels.^45^

Here, we have measured information at one particular step in the visual pathway, vesicles released from bipolar cells, whereas previous work has focused on information carried by spikes in RGCs.^15,21,22,46^ It would be good to understand the relationship between these steps in more detail but, at the moment, simultaneous measurements of release and spikes are not possible. An important feature of the ribbon synapses of bipolar cells is that they encode contrast through changes in both the rate of vesicle fusion and the amplitude of multivesicular events in which two or more vesicles are released in a coordinated manner.^24,46^ Modeling suggests that multivesicular release will enhance the synaptic transfer of information in RGCs with short time constants and reliable synaptic inputs when less convergence is required to trigger spikes.^46^ Contrast is encoded in the activity of most retinal neurons, but it will also be interesting to investigate how information about speed and direction of motion is transferred at synapses and how modulation of this transfer impacts the OMR.

It will be important to identify the sources of noise that limit daylight vision. Several aspects of the variability in the signal leaving bipolar cells have been shown to be under diurnal control, including spontaneous events, the number of vesicles released by a stimulus, jitter in the timing of release events, and variations in the amplitude of multivesicular events.^25^ Less clear is how much of this variability is intrinsic to the bipolar cells and how much is inherited from the signal that bipolar cells receive from cones and the inhibitory synapses of amacrine cells that bombard their synaptic compartments.^13–15^ Neither do we know whether there are diurnal variations in noise from these other sources.

### Investigating the plasticity of visually driven behavior

Single-cell methods such as electrophysiology or calcium imaging can reveal neural patterns correlated with behavior, but interventional approaches such as optogenetics, chemogenetics, or pharmacology provide the strongest evidence for causality. Here, we have used two manipulations. First, intrinsic changes in the state of the retina during the day and, second, drugs that act on endogenous neuromodulatory systems. An advantage of this approach over, for instance, optogenetics, is that it perturbs the circuit across *all* the locations at which the receptors for these neuromodulators normally act in response to changes in the external environment or internal state of the animal.^3^ The fact that we might not yet understand all these actions is not an immediate limitation because information measures do not make any assumptions about the biological mechanisms that alter the information measured at any given point in the circuit. In principle, manipulating retinal function through receptors for neuromodulators could also be used to investigate the plasticity of other visually driven behaviors, such as prey capture or the escape response,^47,48^ which are dependent on the animal’s state of arousal and satiety.^2,49,50^

The OMR is an innate response, but learned visual behaviors in zebrafish, such as fear responses, also vary diurnally, although not necessarily through adjustments in the retina.^51^ This is not surprising, given that different brain regions can generate their own intrinsic rhythms^31^ and about 17% of genes are under circadian control.^52^ A similar scenario is found in mice performing a dim light detection task in a water maze; performance varies diurnally but the output from the most light-sensitive ganglion cells does not.^53^ The change in behavior is instead determined by higher-order processes, including experience-based switches in search strategy.

### Diurnal changes in retinal function in relation to energy consumption

The increased energetic cost of improving performance of the OMR in the afternoon was offset by a 2-to 4-fold increase in the efficiency with which bipolar cells transmit information to the inner retina (Figure 7). Crucially, this change was observed across a range of synaptic release rates and represented an effect over and above the fall in efficiency as release rates increased. The mechanisms that account for this shift in the operating regime of the retina remain to be discovered: genes under circadian control include those coding for cone opsins and various components of the phototransduction pathway.^54^ Currently, we have a limited understanding of the circadian control of ion channels in the retina neuromodulator systems and ion channels in the retina.

The most dramatic change in retinal function in larval zebrafish is observed at night, when visually driven behavior stops and the input to the circuit from cone photoreceptors is almost completely absent.^28^ The shut-down of vision is caused by decreased light-sensitive currents in the outer segment of cones and the disassembly of synaptic ribbons that support transmission of signals to bipolar cells. This form of plasticity might reflect a need to prioritize reduced energy consumption over vision at night,^55^ in much the same way that ribbon synapses of photoreceptors dismantle in hibernating mammals.^56^ A larval zebrafish is in a particularly precarious state of energy balance because it has finished its supply of yolk but is growing rapidly. The behaviors most important for providing energy for growth, foraging and prey-capture,^2,55^ depend on daylight vision because the strike zone that “locks-on” to prey such as paramecia is occupied almost exclusively by UV cones.^26,29,38^ Improving contrast sensitivity in the afternoon might also align with the availability of food, with paramecia becoming more active during the day as water temperatures rise.

Hunger also acts on visual circuits involved in the perception of food; when food deprived, zebrafish’s decisions shift from avoiding to approaching nearby moving objects by recruiting additional prey-responsive neurons in the tectum.^51^ This behavior is modulated by signals from the hypothalamic-pituitary-interrenal axis and the serotonergic system but it is not known whether these signals feed back to the retina. Modulation of visual circuits according to the state of satiety has also been observed in blowflies, where food deprivation suppresses the OMR,^57^ and in mouse visual cortex, where it reduces synaptic ATP use by 29%.^58^ The retina consumes about 50% more oxygen per unit of mass compared with the cortex,^59^ and its size in larval zebrafish suggest that it consumes significantly more energy than the whole of the rest of the animal’s brain. A significant fraction of this energy is used to extrude calcium from synapses through ATP-dependent pumps.^19,60^ The timing of diurnal changes in retinal function, from shut-down of vision at night to peak contrast gain in the afternoon, might therefore represent adaptations that maximize food intake relative to the energetic costs of vision.^61^

## Resource Availability

### Lead contact

Further information and requests for resources and reagents should be directed to, and will be fulfilled by, the lead contact, Leon Lagnado (l.lagnado@sussex.ac.uk).

### Materials availability

This study did not generate new unique reagents.

## Star★Methods

Detailed methods are provided in the online version of this paper and include the following:

- KEY RESOURCES TABLE
- EXPERIMENTAL MODEL
  ○ Zebrafish
  ○ Transgenic animals
- METHOD DETAILS
  ○ Behavioural setup and experiments
  ○ Quantification of swim kinematics
  ○ Multiphoton imaging *in vivo*
  ○ Drug injections
  ○ Analysis sequence for the quantal decomposition of iGluSnFR signals
  ○ Calculations based on Information Theory
- STATISTICAL ANALYSIS

## Star★Methods

### Key Resources Table

**Table:** 

REAGENT or RESOURCE	SOURCE	IDENTIFIER
Chemicals, peptides, and recombinant proteins		
2-amino-6,7-dihydroxy 1,2,3,4-tetrahydronapthalene	abcam	Cat No. ab120150
L-733,060 hydrochloride	Tocris	Cat. No. 1145
Substance P	Tocris	Cat. No. 1156
SCH 23390 hydrochloride	Tocris	Cat. No. 0925
α-Bungarotoxin	Tocris	Cat. No. 2133
1-PHENYL-3-PROPYL-2-THIOUREA	Sigma	Cat. No. S615218
Experimental models: Organisms/strains		
*Wild-type AB zebrafish*	Laboratory of Leon Lagnado	N/A
*Tg(−1.8ctbp2:Gal4VP16_BH)*	Laboratory of Leon Lagnado	Zebrafish Ribeye:Gal4 driver line
*Tg(9xUAS:iGluSnFR_MH)*	Laboratory of Leon Lagnado	Zebrafish iGluSnFR reporter line
*Casper*	Laboratory of Leon Lagnado	Zebrafish homozygous for the roy;nacre double mutant
*Tg(-1.8ctbp2:SyGCaMP6)*	Laboratory of Leon Lagnado	Zebrafish expressing SyGCaMP6 in retinal bipolar cells
*Tg (isl2b:mGCamP6f)*	Laboratory of Tom Baden	Zebrafish expressing mGCamP6f in retinal ganglion cells
Recombinant DNA		
Tol2 pDest 9 x UASiGluSnFR	Laboratory of Michael Orger, Champalimaud, Lisbon.	iGluSnFR plasmid
Ribeye SyGCaMP6.10.50 0_Bleeding heart	Laboratory of Loren Looger, UCSD.	SyGCaMP6f plasmid
Software and algorithms		
ScanImage	MBF Bioscience	ScanImage v.3.6
IgorPro	Wavemetrics	IgorPro v.8
GlueSniffer	James et al.^24^	https://github.com/lagnadoLab/glueSniffer
Bonsai	Bonsai Foundation	https://bonsai-rx.org
BonZeb	Nicholas Guilbeault	https://ncguilbeault.github.io/BonZeb/

### Experimental Model

#### Zebrafish

Zebrafish were raised and maintained under standard conditions on a 14 h light/10 h dark cycle.^62^ The composition of the E2 medium in which embryos were kept and experiments were carried out was as follows: Na2HPO4 0.05 mM, MgSO4 1 mM, KH2PO4 0.15 mM, KCl 0.5 mM, NaCl 15 mM. CaCl 1 mM, NaHCO3 0.7 mM, pH 7.0–7.5. For behavioural experiments, we used 6-8 days post fertilization (dpf) AB wild type larvae. For imaging experiments, heterozygous or homozygous hypopigmented *casper* larvae of the same age were used to aid imaging. Transgenic fish were treated with1-phenyl-2-thiourea (200 μM final concentration; Sigma) from 10 hours post-fertilization to reduce pigmentation. PTU reduces pigmentation and is an inhibitor of tyrosinase,^63^ but at the concentration used in this study endogenous dopamine levels in the retina remain functionally significant,^25^ as shown by the effects of a D1 receptor antagonist in the afternoon (Figures 4B, 5A, and 5B). All animal procedures were performed in accordance with the Animal Act 1986 and the UK Home Office guidelines and with the approval of the University of Sussex Animal Welfare and Ethical Review Board.

#### Transgenic animals

Imaging experiments were carried using the following transgenic lines of zebrafish:

i) *Tg(–1.8ctbp2:Gal4VP16_BH)* fish that drive the expression of the transcriptional activator protein Gal4VP16 were generated by co-injection of I-SceI meganuclease and endofree purified plasmid into wild-type zebrafish with a mixed genetic background. A myocardium-specific promoter that drives the expression of mCherry protein was additionally cloned into the plasmid to allow for phenotypical screening of founder fish.
ii) *Tg(9xUAS:iGluSnFR_MH)* fish driving the expression of the glutamate sensor iGluSnFR under the regulatory control of the 9 x UAS enhancer elements were generated by co-injection of purified plasmid and tol2 transposase RNA into offspring of AB wild-type fish outcrossed to casper wildtype fish. The sequences for the myocardium-specific promoter driving the expression of enhanced green fluorescent protein (mossy heart) were added to the plasmid to facilitate the screening process.
iii) *Tg(–1.8ctbp2:SyGCaMP6)* fish were generated by co-injection of I-SceI meganuclease and endofree purified plasmid into wild-type zebrafish with a mixed genetic background. The GCaMP6f variant (alternative name GCaMP3 variant 10.500) was kindly provided by L. Looger (Janelia Farm). This variant holds a T383S mutation in comparison to the commercially available GCaMP6-fast version (Addgene plasmid 40755).
iv) *Tg (isl2b:mGCamP6f)* was kindly provided by Tom Baden (University of Sussex).^64^

### Method Details

#### Behavioural setup and experiments

Larvae swam freely in a clear 6 cm diameter and ~3 mm depth watch glass filled with E2 solution. The watch glass was fixed on a custom 3D built support that was attached to a 10 cm diameter Petri dish. The support fit tightly with the watch glass edge so to obscure the sidewall reflections that could confound the fish detection algorithm. This swimming arena was illuminated by an infrared (IR) light-emitting diode (810 nm) from below. Visual stimuli were projected using a DLP projector (Vamvo, Shenzhen, China) onto a diffusing screen on the bottom of the swimming arena after reflection by 5 cm diameter cold mirror (Thorlabs). With the exception of the projector, all these components were enclosed in a light-proof box with a 25 mm aperture for the projected light enter the box. The temperature inside the box was set at 27^◦^C through a temperature controller (Ringder AC-112) connected to two heating mats attached to the side walls.

Fish behaviour was recorded at 150 Hz using a high-speed, monochrome and IR sensitive camera (Omron Sentech STC-CMB200PCL-NIR, Alrad Instruments Ltd, UK) connected to a digital framegrabber (Euresys Grablink Full XR, Stemmer Imaging, Germany). A Navitar zoom lens (Thorlabs, Germany), used in conjunction with an IR pass filter (Thorlabs) was attached to the camera to image the swimming arena. Larvae were transferred directly from the incubator (where the circadian cycles were set) into the behavioural chamber where they swam ~5 mm from the diffuser screen where the stimulus was presented. The spatial frequency of the grating was 2 cm, which corresponds to 0.007 cycles per degree. The dendritic trees of bipolar cells are 10-70 μm in diameter^65^ so the fluctuations in intensity experienced by any individual bipolar cell were close to spatially uniform at this very low spatial frequency. A total of 318 zebrafish larvae were used in behavioural experiments.

Bonsai^66^ workflows, supplemented by Bonzeb^67^ packages, were programmed with a background-subtracted algorithm to detect and to extract the online position and orientation of the fish. The Bonsai workflows used this information to render and update the visual stimulus in real time and provide consisted motion stimulation relative to the fish body axis. At the start of each trial, background subtraction was first calculated by inducing fish to swim upon presentation of stepwise gratings moving at a random direction. The trial then began in which sinusoidal gratings drifted parallelly relative to the fish body axis and were locked in close loop to the fish orientation. Left- and right-wards drifts were balanced in all experiments. The relation between pixel value and luminance of the projector was measured with a photodiode and non-linearities corrected for in the Bonsai script controlling the visual stimulus. In CRF experiments, a trial consisted in the presentation of the moving gratings with a temporal frequency of 1 Hz at a pre-set contrast level for 30 or 60 seconds. CRFs were described as Hill functions of the form *R* = *R*_max_*(*S*^h^/(*S*^h^ + *S*_1/2_^h^)), where *R* is the response, *S* is the stimulus, *h* is the Hill coefficient and *S*_1/2_ is the stimulus generating the half-maximal response. In trial-based experiments, eleven different contrasts between 15 and 35% were presented pseudo-randomly at a temporal frequency of 5 Hz. Each contrast presentation lasted 2 s and each contrast was presented 100 times.

#### Quantification of swim kinematics

Offline behavioural analysis was performed using custom IgorPro scripts (WaveMetrics, Lake Oswego, USA). The frame-by-frame position of the fish was used to derive their swim speed throughout the trial. Individual swimming bouts were then extracted by peak detection from the speed traces. The start and end of a swimming bout was defined by calculating a threshold value for each trial based on the Gaussian fit of the distribution of all velocities extracted on that trial. The start-end of each swimming bout was then superimposed on the fish orientation trace to calculate the turning angle in that event. Turning angles were measured in radians with positive values assigned to turns in the direction of the presented bar drift. The turning speed was calculated by dividing the final cumulative angle (the cumulative value of all the turns at the end of the stimulus presentation) by the duration of the trial. For all behavioural graphs, error bars represent standard error of the mean across fish.

#### Multiphoton imaging *in vivo*

Experiments were carried out on a total of 154 zebrafish larvae. Fish were immobilized in 3% low melting point agarose (Biogene) in E2 medium on a glass coverslip and mounted in a chamber where they were perfused with E2. Imaging was carried out using a two-photon microscope (Scientifica, Uckfield, UK) equipped with a mode-locked titanium-sapphire laser (Chameleon, Coherent) tuned to 915 nm and an Olympus XLUMPlanFI 20x water immersion objective (NA 0.95). To prevent eye movements, the ocular muscles were paralyzed by injection of 1 nL of α-bungarotoxin (2 mg/mL) (Tocris) behind the eye. Most imaging was carried out in the dorsal retina. The signal-to-noise ratio of the microscope was optimized by collecting photons through both the objective and an Olympus, NA 1.4 sub-stage oil condenser. Emission was filtered through GFP filters (HQ 535/50, Chroma Technology) before detection with GaAsP photomultipliers (H7422P-40, Hamamatsu). The signal from each detector passed through a current-to-voltage converter and then the two signals were added by a summing amplifier before digitization. Scanning and image acquisition were controlled under ScanImage v.3.6 software. For iGluSnFR recordings, images were acquired at 10 Hz (128 × 100 pixels per frame, 1 ms per line) while linescans were acquired at 1 kHz. In SyGCaMP and mGCamP6f recordings images were acquired at 20 Hz (128 × 50 pixels per frame, 1 ms per line).

The microscope was synchronized to visual stimulation which consisted in full-field light stimuli generated by an amber LED (*l*_max_ = 590 nm, Thorlabs), filtered through a 590/10 nm BP filter (Thorlabs), and delivered through a light guide placed close to the eye of the fish. These wavelengths will most effectively stimulate red and green cones but do not stimulate UV cones most common in the strike zone.^29^ The light intensity varied sinusoidally with a temporal frequency of 5 Hz around a mean in the low photopic range (~400 nW mm^-2^). About 60% of incident light reaches the retina of a larval zebrafish,^38^ so this converts to an isomerization rate of ~180 R* s^−1^. We used spatially uniform stimuli in experiments measuring synaptic activity rather than projecting a grating on the bottom of the dish because the latter did not allow for use of a substage condenser to improve the SNR of imaging. Individual bipolar cells also experience spatially uniform stimuli in the center of their receptive fields when assaying the OMR (see above).

Relative changes in fluorescence (ΔF/F) during a stimulus were measured relative to the baseline before the stimulus. Measurements of contrast sensitivity with iGluSnFR were made by stimulating the fish with a series of 2 s stimuli. The distribution of events amplitudes and the temporal precision were measured during 30 s stimulation at a given contrast.

No statistical methods were used to predetermine sample sizes. Experiments were repeated until trends in results were clear and this resulted in sample sizes at least equivalent to previous publications. Different experiments were repeated between 10 and 50 times and only reported if similar results were observed in >95%. Data were only excluded from the analysis if the signal-to-noise ratio (SNR) of the iGluSnFR signals elicited at a given synapse was not sufficient to detect unitary responses to visual stimuli with a SNR of at least three.

#### Drug injections

Substance P was manipulated by injecting the agonist of the NK-1 receptor (NK-1R) Substance P (Tocris) to an estimated final concentration of 20 nM, and the effects of endogenous Substance P were antagonized by injection of L-733060 (Tocris) at estimated final concentration of 20 nM. Dopamine signalling was manipulated by injecting the antagonist of D1 receptor (D1R) SCH 23390 (Tocris) at a final estimated concentration of 20 nM and the long-lasting dopamine receptor ligand [3H] 2-amino-6,7-dihydroxy 1,2,3,4-tetrahy-dronapthalene (ADTN) (abcam) was injected to a final estimated concentration of 200 nM. We confirmed that these drugs gained access to the intravitreal space by including 1 mM Alexa 594 in the injection needle; within 5 min of injection the dye could be detected within the inner plexiform layer of the retina. Vehicle injection did not affect synaptic responses to varying contrast. OMR experiments were performed 5-10 minutes after the drug injection.

#### Analysis sequence for the quantal decomposition of iGluSnFR signals

Estimating a time-series of quantized events from linescans across the synaptic compartment was carried out with custom software that has been described previously.^24,68^ Briefly, the analysis consisted of the following steps:

##### Separation of regions of interest (ROIs) by spatial decomposition

The spatial profile across the compartment was measured as a temporal average of the fluorescence signal along the linescan and this profile fitted with a sum of Gaussians, where each represents a point source corresponding to an active zone.

##### Time series extraction by weighted averaging

Once each spatial component had been defined, a time series for that component, F(t), was computed by fitting the linescan at each time point with a weighted sum of all Gaussian components.

##### Baseline correction and calculation of ΔF/F_0_

The iGluSnFR signal used for all analyses was the relative change in fluorescence, ΔF/F_0_, calculated from the bleach-corrected signals. The most frequent value (that is, the baseline) of the trace was used as F_0_.

##### Identification of events by Wiener deconvolution

Release events within an active zone were identified by their characteristic kinetics using a Wiener filter with kernel h(t) of the following form:

(Equation 1) $$h(t)=A\cdot e^{-t/\tau_{f}}\cdot \left(1-e^{-t/\tau_{r}}\right)$$

where A is the amplitude of the event and τ_r_ and τ_f_ and are the time constants for rise and fall in the signal, respectively. Transients at most synapses could be described using τ_f_ = 0.06 s and τ_r_ = 0.001 s. The result of the Wiener deconvolution was a time series in which glutamate release events were described approximately as impulses of varying amplitudes.

##### Extraction of events

Although the use of Wiener deconvolution significantly improved the SNR, it was still necessary to set a threshold to distinguish events from noise, which was usually 3–4 standard deviations above the baseline.

##### Amplitude clustering and quantal time series

Based on the evidence that glutamate transients of varying amplitude were integer multiples of a unitary event or quantum^24^ we partitioned events into numbers of quanta using a maximum likelihood estimate. Defining a time series as the number of quanta within each event allowed for computation of vesicle release rates and information theoretic measures.

#### Calculations based on Information Theory

The methods we used to quantify the information transmitted at individual synapses have been described previously.^24,25^ Briefly, the time series of events was converted into a probability distribution using time bins of 20 ms, so that each bin contained either zero events or one event of an integer amplitude. We then counted the number of bins containing events of amplitude 0, 1, 2, 3 etc. By dividing the number of bins of each type by the total number of bins for each different stimulus, we obtained the conditional distribution of **Q** given **S**, *p* (***Q***|***S***), where **Q** is the random variable representing the *quanta/bin* and **S** is the random variable representing the *stimulus contrasts* presented throughout the course of the experiment. A uniform distribution of eleven contrasts was used ranging ±10% around C_1/2_, which was first estimated for each synapse. The joint probability distribution by the chain rule for probability (given the experimentally defined uniform distribution of stimuli **S**):

(Equation 2) p(S,Q)=p(Q∣S)p(S)

To convert this distribution into the conditional distribution of S given Q, we used the definition of the conditional distribution:

(Equation 3) $$p(S|Q)=\frac{p(S,Q)}{p(Q)}$$

From these distributions we computed the mutual information I(**S;Q**)^69^ as:

(Equation 4) I(S;Q)=H(S)−H(S∣Q)

representing the amount of information observing each quantal event type q ϵ **Q** carries about the stimulus distribution **S**.

## Statistical Analysis

All data are given as mean ± s.e.m. unless otherwise stated in the figure legends. All statistical tests met appropriate assumptions and were calculated using inbuilt functions in IgorPro (Wavemetrics) and SPSS 29 (IBM). Significance was defined as p < 0.05. Data collection was not randomized because all experiments were carried out within one set of animals. Delivery of different stimuli was randomized where appropriate.

## Data Availability

All data and code reported in this paper will be shared by the lead contact upon request. This paper does not report original models. Any additional information required to reanalyze the data reported in this paper is available from the lead contact upon request.

## In brief

Simões et al. quantify how diurnal modulation of the retina alters the optomotor response of zebrafish. Behavioral contrast gain scales with the rate at which synapses of bipolar cells transmit contrast information. Diurnal increases in the information efficiency of these synapses offsets the energetic cost of enhanced behavioral performance.

## Highlights

- Diurnal modulation of retinal function alters the gain of the optomotor response
- Behavioral gain scales with bipolar cell synaptic information rates
- Retinal noise limits behavioral performance under photopic conditions
- Diurnal increases in retinal information efficiency offset cost of improved behavior

## Footnotes

**Supplemental Information**
Supplemental information can be found online at https://doi.org/10.1016/j.cub.2025.07.007.
